# A multi-species comparative structural bioinformatics analysis of inherited mutations in α-D-Mannosidase reveals strong genotype-phenotype correlation

**DOI:** 10.1186/1471-2164-10-S3-S33

**Published:** 2009-12-03

**Authors:** Javed Mohammed Khan, Shoba Ranganathan

**Affiliations:** 1 Department of Chemistry and Biomolecular Sciences and ARC center of excellence in Bioinformatics, Macquarie University, NSW 2109, Australia; 2 Department of Biochemistry, Yong Loo Lin School of Medicine, National University of Singapore, 8 Medical Drive, Singapore 117597

## Abstract

**Background:**

Lysosomal α-mannosidase is an enzyme that acts to degrade N-linked oligosaccharides and hence plays an important role in mannose metabolism in humans and other mammalian species, especially livestock. Mutations in the gene (*MAN2B1*) encoding lysosomal α-D-mannosidase cause improper coding, resulting in dysfunctional or non-functional protein, causing the disease α-mannosidosis. Mapping disease mutations to the structure of the protein can help in understanding the functional consequences of these mutations and thus indirectly, the finer aspects of the pathology and clinical manifestations of the disease, including phenotypic severity as a function of the genotype.

**Results:**

A comprehensive homology modeling study of all the wild-type and inherited mutations of lysosomal α-mannosidase in four different species, human, cow, cat and guinea pig, reveals a significant correlation between the severity of the genotype and the phenotype in α-mannosidosis. We used the X-ray crystallographic structure of bovine lysosomal α-mannosidase as template, containing only two disulphide bonds and some ligands, to build structural models of wild-type structures with four disulfide linkages and all bound ligands. These wild-type models were then used as templates for disease mutations. All the truncations and substitutions involving the residues in and around the active site and those that destabilize the fold led to severe genotypes resulting in lethal phenotypes, whereas the mutations lying away from the active site were milder in both their genotypic and phenotypic expression.

**Conclusion:**

Based on the co-location of mutations from different organisms and their proximity to the enzyme active site, we have extrapolated observed mutations from one species to homologous positions in other organisms, as a predictive approach for detecting likely α-mannosidosis. Besides predicting new disease mutations, this approach also provides a way for detecting mutation hotspots in the gene, where novel mutations could be implicated in disease. The current study has identified five mutational hot-spot regions along the *MAN2B1 *gene. Structural mapping can thus provide a rational approach for predicting the phenotype of a disease, based on observed genotypic variations.

## Background

α-D-mannosidase is a lysosomal enzyme which is involved in the catabolism of N-linked glycoproteins through the sequential degradation of high-mannose, hybrid and complex oligosaccharides [1]. The deficiency of this enzyme results in a recessively inherited lysosomal storage disease, called α-mannosidosis, which has been observed in different species in the animals, including domestic cows (*Bos taurus*), cats (*Felis catus*), guinea pigs (*Cavia porcellus*), sheep (*Ovis aries*) and in humans (*Homo sapiens*). It was first characterized in humans by Oeckermann in 1967 [2]. Mutations in the *MAN2B1 *gene, located on chromosome 19 (19 p13.2-q12), encoding lysosomal α-D-mannosidase cause improper coding resulting in dysfunctional or non-functional protein and hence causing the disease. Characterized by immune deficiency, facial and skeletal abnormalities, hearing impairment, and intellectual disability, α-mannosidosis occurs in 1 of 500,000 live births [2]. However, clinicians, geneticists and molecular biologists have not been able to correlate the genotypic mutations with the observed phenotype [2].

Mapping disease mutations to the structure of a protein can help in understanding the finer aspects of the pathology and clinical manifestations of a disease. Although restricted to diseases where the protein concerned has a known 3-D structure, such an approach is adequately detailed at the molecular level to provide rational explanation for the pathological role of mutations, using protein 3D structure (SOX9 [3]; human factor H [4,5]). Therefore, we have attempted a structural bioinformatics approach to understand the role of the different mutations causing α-mannosidosis with differing phenotypes.

From OMIM (Online Mendelian Inheritance in Man) [6], OMIA (Online Mendelian Inheritance in Animals) [7] and published literature [8], a list of inherited mutations for α-mannosidosis has been identified. Various mutations like missense, nonsense, insertions, deletions and also some splicing mutations have been described in the four species to date. Of these only the missense mutations result in a substitution in the protein sequence and were modeled to study their effect on the phenotype. All the other mutations result in the truncation of the protein and its improper function.

An X-ray crystal structure for bovine lysosomal α-D-mannosidase [9] (PDB ID: 1O7D), solved at a reasonably good resolution of 2.7 Å, is available, albeit lacking two vital disulfide bonds, that hold the five protein chains of the mature α-D-mannosidase protein together, as well as nine of the 20 ligands and a few structurally and functionally important residues. To overcome the limitations of the available 3D structure, we have used homology modeling approaches to reconstruct the complete lysosomal α-D-mannosidase for human, bovine, cat and guinea pig, to which structures we have then mapped all known mutations. The truncation mutations, with the exception of a single truncation in cat, were not modeled, as most of them produced proteins spanning less than two of the five protein chains, leading to a completely compromised active site.

Our comprehensive analysis, taking into account all non-splicing mutations (see Additional file 1: table S1) known to cause α-mannosidosis, reveals a strict genotype-phenotype correlation, contrary to the reports of Malm and Nilssen [2] and Lyons *et al. *[8]. This disease can be comparatively well studied as it occurs in different species, providing us with an evolutionary basis for the conserved regions of the protein sequence, as well as active site conservation, where mutations could result in disastrous consequences. Regions with several mutations represent hot-spots where novel mutations could lead to disease. Based on the location of these hot-spots vis-à-vis the active site of the protein, it is also possible to predict the disease phenotype: mild, moderate or severe, extrapolating from known mutations and disease phenotypes. In this paper, we describe a prototype structural bioinformatics analysis method applied to α-mannosidosis, which can be extended to several other diseases for predicting novel disease phenotypes and for developing therapies as well as designing drug/inhibitor molecules.

## Materials and methods

Observations that the structure of proteins is better preserved during evolution than their sequence, have lead to homology modeling [10,11] as an reliable methodology for generating 3D structural models of proteins, when the structure of a homologue is available. The most critical step in modeling a correct structure is the alignment of the target sequence with that of the template structure. 3D models of the complete mature wild-type (WT) bovine α-D-mannosidase protein were developed and put through a series of checks for structural verification and analysis to select the best structural model. From this bovine model structure, homology models were constructed for the WT proteins for the other species.

### Sequence retrieval

Four complete WT lysosomal α-mannosidase amino acid sequences were retrieved from the Swiss-Prot database [12], one each, for bovine (Accession No: Q29451), human (Accession No: O00754), guinea pig (Accession No: Q8VHC8) and cat (Accession No: O46432).

### Sequence alignment

Full-length WT protein sequences, spanning all five protein chains, were aligned to the sequence corresponding to the PDB structure, 1O7D, using ClustalX [13] with default BLOSUM scoring matrices. As the signal peptide (consisting of about 50 N-terminal amino acids) is not present in the mature protein, these were removed from the WT sequences, prior to alignment. The gaps in the alignment were carefully scrutinized and edited manually to preserve chain boundaries and the conservation of structurally and functionally important residues, especially in view of the large segments of residues missing in the PDB structure, 1O7D.

### Molecular modeling

Since α-D-mannosidase is cleaved into five chains, which assemble into a functional enzyme, MODELLER 7V7 [14] proved to be the only homology modeling program allowing multiple chain modeling with ligand inclusion. The models are constructed by optimally satisfying spatial constraints and dihedral angle restraints derived from the alignment of the template structure with the target sequence [15] and from the CHARMM-22 [16] force field which together enforce proper stereochemistry. Three structural models were generated for each WT and mutated sequence. The model with the lowest current energy and objective function was selected for analysis after carrying out quality assessment and structural refinement.

**Figure 1:**
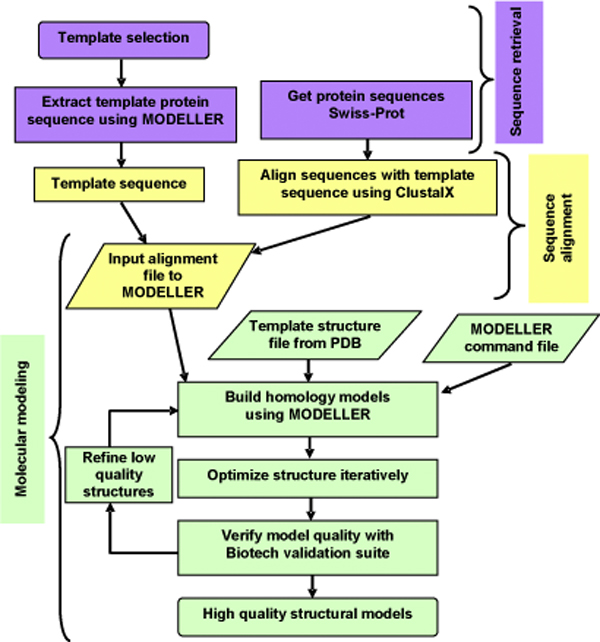
**Flowchart of the three-step modeling procedure used in this study**. The three main steps involved in building a structural model are described, with reference to query sequence, template sequences and template structure.

**Figure 2:**
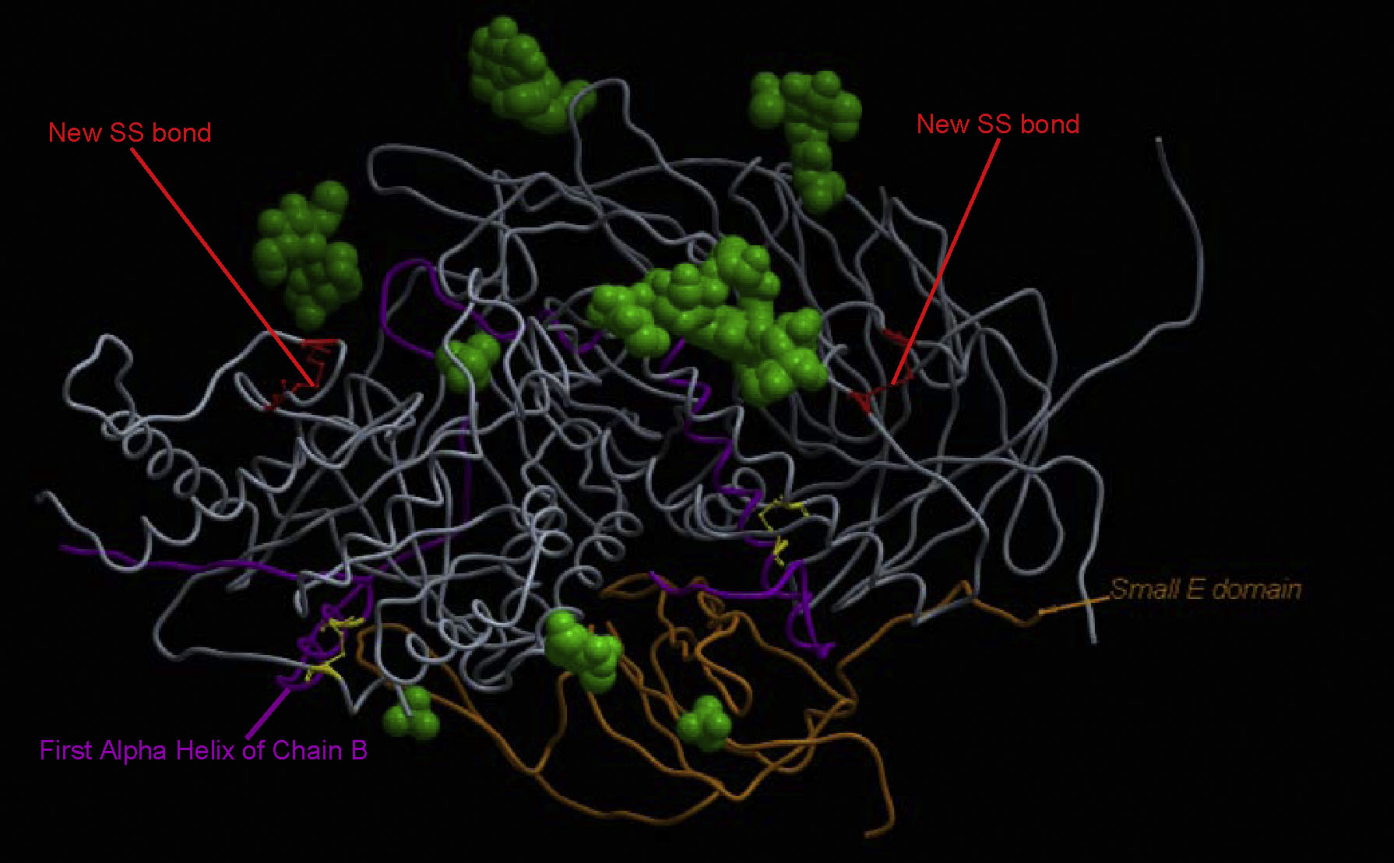
**Structural model of wild-type bovine α-mannosidase**. The structure is shown in Cα trace representation (in grey) with chains B and E highlighted in magenta and gold respectively. The bound ligands present in the template structure are shown as green spheres. Disulfide bonds in the template structure are in yellow ball-and-stick representation, with new disulfide bonds in the model in red.

A flowchart of the three-step procedure, each involving various sub-steps, used in this study is depicted in Figure 1. Swiss-Prot has annotated the existence of four disulfide bridges and 11 ligands. However, the template structure contains only two disulfide bridges and 11 ligands, respectively. We have now rebuilt the two missing disulfide bridges along with all the existing ligands (shown in Figure 2) in our mannosidase models in order to analyze the effect of the observed mutations on all four disulfide bridges and binding of the 11 ligands. The positions of these 11 ligands were extrapolated from the template structure into all the structural models using MODELLER. After high-quality WT structural models for all four species were generated, they were used as templates to build models corresponding to all the substitution mutations in three of the species (bovine, human and guinea pig). The feline mutation results in a truncation at codon 645 of the *MAN2B1 *gene, translating which we obtained the protein sequence, selecting the frame with methionine as the first residue, for model building.

**Figure 3:**
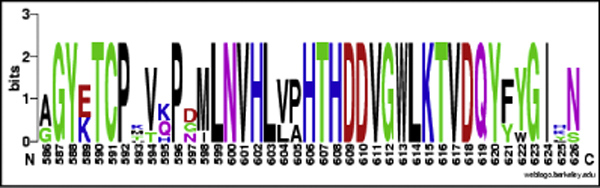
**WEBLOGO alignment of putative *MAN2B1 *pro-peptide sequences from six mammalian species**. The alignment represents sequences from human, bovine, cat and guinea pig, along with two other mammalian species, mouse and macaque.

Swiss-Prot also classified a potential pro-peptide (591-621) within the bovine WT α-D-mannosidase sequence. These residues were found to be present in the template structure (1O7D) and the WEBLOGO [17] representation of alignment of the six mammalian lysosomal α-mannosidase sequences (Figure 3) shows this region to be fairly well conserved. Hence, the putative pro-peptide region was retained in all the structural models. The template structure contained 39 water molecules, mostly present on the surface as water molecules of crystallization. Of the internal water molecules, none were within 4 Å of the Zn and Tris ligands and thus were unlikely to be functionally critical. All water molecules were therefore removed from the template structure and were excluded from this analysis.

The model structures were visualized using ICM [18]. The quality of each structural model was evaluated using the three major structural assessment tools, PROCHECK [19], WHAT IF [20] and PROVE [21], which together perform checks on stereo-chemical quality, residue geometry, bond-angle, bond-length and volumetric analysis. These three programs are available as part of the Biotech Validation Suite for Protein Structures.

## Results and discussion

### Assessment of the WT structural models

MODELLER generates structural models from a pool of randomized potential starting conformations, resulting from multiple iterations of stereo-chemical refinements. The structural quality of the final models was assessed using the Biotech Validation Suite for Protein Structures as described earlier. Table 1 summarizes the results of various quality checks that were performed on the WT models, namely Bwt, Hwt, Gwt and Fwt, for bovine, human, guinea pig and cat sequences, respectively. All models have an RMSD score between 0.643-0.894 Å which is considered excellent for homology models. The WHATIF *Z*-score (1.310-1.500) for all the models was also better than the default of 1.55 for a high-quality structure, and the PROVE *Z*-score average (from -0.04 to 0.08) is well within -0.10 to 0.10 for a well-resolved structure. Most importantly, all the WT models had 98.2% - 98.9% of all their residues in conformationally permitted regions as predicted by PROCHECK, with 85% being the minimum requirement for high-resolution X-ray crystallographic structures. The high degree of similarity between the target sequences and the template along with strict modeling protocols adopted in this study, have contributed to the generation of high quality WT models.

**Table 1:** Structural quality assessment for the wild-type models

Models	PROVE	PROCHECK		WHATIF	**RMSD (Å**)
		
	*Z*-Score average	Residues in most favoured regions	Residues in allowed regions	*Z*-Score	
Bwt	0.08	89.9%	8.8%	1.31	0.70
Hwt	-0.04	89.1%	9.7%	1.34	0.64
Gwt	0.05	86.7%	11.5%	1.50	0.89
Fwt	0.05	89.0%	9.9%	1.40	0.71

### Mapping mutations to WT structural models

**Figure 4:**
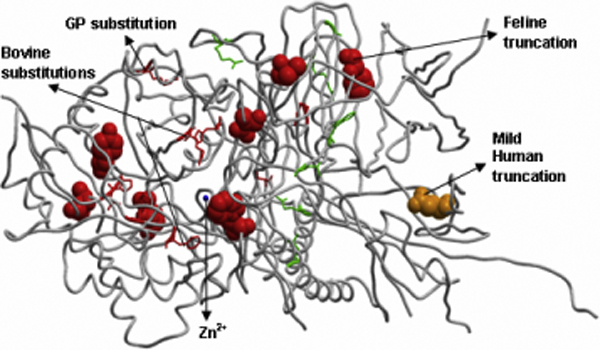
**Structural model of WT bovine α-mannosidase showing the location of all mutations studied in different species**. The spheres denote positions of truncation mutations. Amino acids involved in substitution mutations are shown in stick representation. The catalytic zinc ion is shown as a blue sphere. Mutations are coloured red, green and orange, representing harmful, viable and mild phenotypes, respectively.

We have primarily constructed a mutation map, by mapping available mutations in the context of the enzyme active site to the structural models of α-mannosidase, based on multiple sequence alignment of all WT sequences and the sequence of the 1O7D structure (not shown), to understand where the observed mutations occur (Figure 4 shows the bovine model). We note that most of the mutations with lethal phenotypes are located in and around the active site, thereby affecting the functionality of the enzyme. Based on the analysis of structural models, we have correlated the position and functional consequence of each mutation to the observed phenotypic consequence. Table 2 suggests that the mutations close to active site have a direct effect on the enzymatic function of the protein leading to lethal phenotypes. Where as mutations which act to destabilize the fold, in turn, affect the active site, thereby resulting in harmful disease phenotypes. Mutations distant to the active site cause minimal or no damage to the structure and function of the enzyme, resulting in mild or viable genotypes and phenotypes. These three phenotypes correspond to Type 3, Type 2 and Type 1 clinical phenotypes described by Malm and Nilssen [2].

**Table 2:** Correlation of the effect of substitution mutations on folding and observed disease phenotype. Mutations leading to severe phenotypes are highlighted in bold font. AS: Active site

S. No	Species	Mutated residues	Structural location	Effect on Structure	Phenotypic effect
1.	Human	**H72L **[25]	**In AS**	**Disrupts AS**	**Lethal** (Type 3)
		
		**H200N **[24], **T355P **[25], **P356R **[25,27], **E402K **[25]**, S453Y **[26]	**Close to AS**	**Destabilizes the fold**	**Harmful** (Type 2)
		
		W714R [25], R750W [25,27], G801D [28], L809P [25]	Away from AS	Minor perturbation	Mild or Viable (Type 1)

2.	Bovine	**R220H **[21]	**In AS**	**Disrupts AS**	**Lethal** (Type 3)
		
		**F320L **[21]	**Close to AS**	**Destabilizes the fold**	**Harmful** (Type 2)

3.	Guinea pig	**R227W **[29]	**Close to AS**	**Destabilizes the fold**	**Harmful** (Type 2)

#### Substitutions

**Figure 5:**
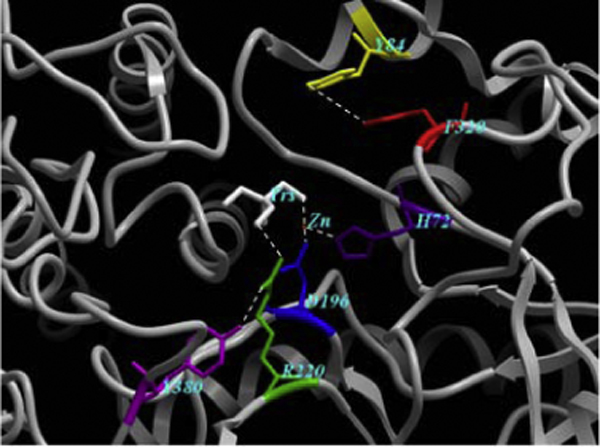
**Part of the enzyme active site, with a high concentration of mutations leading to lethal phenotypes**. Several aromatic residues surround the active site and potentially are involved in binding of the Tris ligand. Tris (white) is held in position by the catalytic nucleophile, D196 (blue) *via *a zinc ion (orange). R220 (green) is directly involved in ligand binding, along with H72 (purple) and Y380 (magenta). The aromatic side chains of F320 (red) and Y84 (yellow) interact to hold the structure together. These interactions are shown in dotted lines.

Two mutations (R220H and F320L in Table 2) have been reported to cause fairly severe α-mannosidosis in cattle [22]. R220H (Figure 4) is due to a G<A change in the nucleotide sequence at position 662. It reduces the enzyme activity to 2% [22] by affecting the orientation and stability of the catalytic nucleophile (D196) [23] causing impaired substrate binding. Analysis of our structural model confirms the hypothesis that the mechanism of inactivation by this mutation may affect both substrate binding and its hydrolysis, as H220 can form a hydrogen bond with D196 and Y380 but not with the Tris ligand (1H 2-amino-2-hydroxymethyl-propane-1,3-diol) or the substrate, unlike the WT R220 (Figure 5). F320L (Figure 4) is responsible for reducing a-mannosidase activity in the liver of affected cattle to 0.3% [22]. Figure 5 shows how chain A could be anchored in its current position by an aromatic interaction between the F320 and the nearby Y84 [9,24]. As both residues are on the same chain, this does not inhibit the assembly of the five chain enzyme. However, in the F320L mutant, the non-aromatic leucine residue destroys the ring-stacking interaction between the aromatic side-chains F320 and Y84 in the WT enzyme, thereby destabilizing the 3D structure of the enzyme.

On the whole, 13 substitution mutations have been characterized to cause α-mannosidosis in humans with experimentally verified loss in enzyme activity. H200N [25] and H72L [26] disrupt the active site, while E402K [26] and S453Y [27] lead to disruption of the ionic linkages with the surrounding charged residues that would otherwise stabilize the protein structure. H72L disturbs a metal-coordinating residue (Figure 5). H200N alters substrate binding and other catalytic properties of the enzyme resulting in no residual enzyme activity. T355P and P356R [26,28] are located in first α-helix of chain B (Figure 2). These mutations presumably affect the initiation of the helix and thus are likely to disturb the folding of active-site domain. W714R, L809P, R750W [26,28] and G801D [29] are located in chain D, where they perturb the structure of the enzyme minimally and result in mild/viable phenotypes. L518P and R916S [25] are considered the only exception to our correlation, as they lie away from the active site yet potentially disturb the interaction of small E domain (Figure 2) with active site domain. R916S also damages the hydrogen bonding between R916 and D170. These mutations result in a moderately harmful phenotype. R227W in guinea pigs is due to T<C change in the nucleotide sequence at position 679 causing significant loss in enzyme activity [30]. R227 is a structurally and functionally important conserved residue in all the species and its substitution could affect ligand binding.

#### Truncations

**Figure 6:**
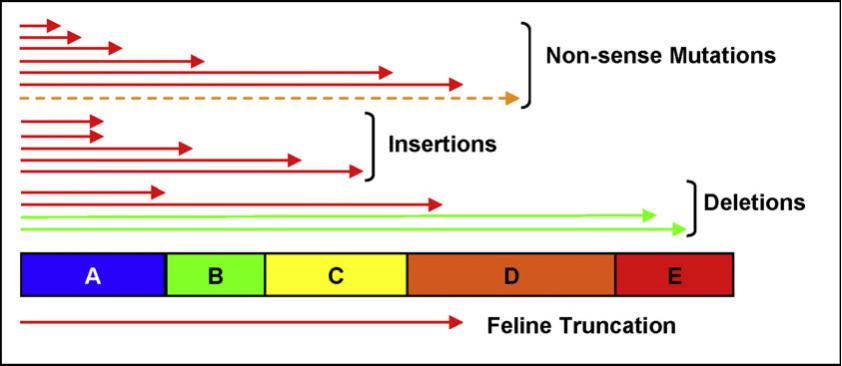
**Mapping truncation mutations to the different chains of the human α-mannosidase protein**. The extent of nonsense, insertion and deletion mutants are shown as red, orange and green arrows, represent lethal, moderate and mild phenotypes, respectively.

Truncations in the human enzyme are due to three different mutation classes, called non-sense mutations, insertions and deletions [26]. They result in proteins with two or three peptide chains (Figure 6), instead of five chains in the WT protein, causing severe damage to the viability of the enzyme to assemble into a functional protein, leading to fatal phenotypes. However, a few truncations lead to viable genotypes with mild phenotypes, as they are located far from the active site (in chains D and E). The feline truncation, 1748del4, represents a 4-base deletion spanning nucleotide positions 1749-1752 of the *MAN2B1 *gene (Figure 6). This causes termination at codon 645 resulting in a truncated protein with only three full-length chains, resulting in impaired binding of the Tris ligand and exhibiting a very severe phenotype in cats (especially in Persian cats) [31].

### Prediction of potentially harmful mutations

**Figure 7:**
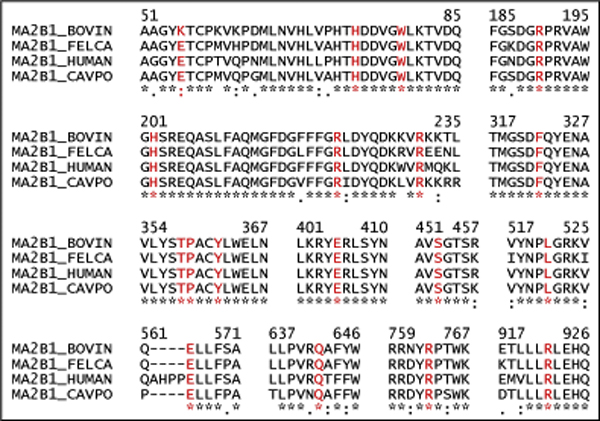
**Conservation analysis of mutations across the four species**. Alignment of the wild-type sequences from all four species shows a high level of sequence similarity, with conserved (*), conservatively substituted (:) and semi-conservatively substituted (.) residues. Sequence segments known to be mutational hot-spots are shown. Sequence names and numbers are from Swiss-Prot.

α-mannosidase has a highly conserved sequence across all species with minor changes to suit the metabolic requirements. As seen in Figure 7, almost all the mutated residues causing fatal/harmful phenotypes are highly conserved, with E53 prevalent in three of the four species studied and replaced by K53 in the bovine sequence. Based on our analysis, the residues H72, E402, H200, S453, T355 and P356, which when substituted cause extremely fatal forms of α-mannosidosis in human, could result in harmful phenotypes in cow, guinea pig and cat, if mutated, due to their highly level of sequential and positional (near the active site) similarity in all the species. Similarly, E53, W77, R188, Y359, E563, Q639 and R760 seem to be extremely conserved throughout, thus mutations at these residues could cause lethal genotypes/phenotypes in cow, guinea pig and cat. It is also evident that R220 and F320, whose substitution results in nil or extremely low enzyme activity in cow, and R227 which when substituted causes improper ligand binding in guinea pig, are conserved across all the species thereby provoking the idea that mutations involving these residues in human and cat could prove to be severe. Due to the different mammalian species represented here, all these positions can be considered potential disease-causing mutations for all mammals.

**Figure 8:**
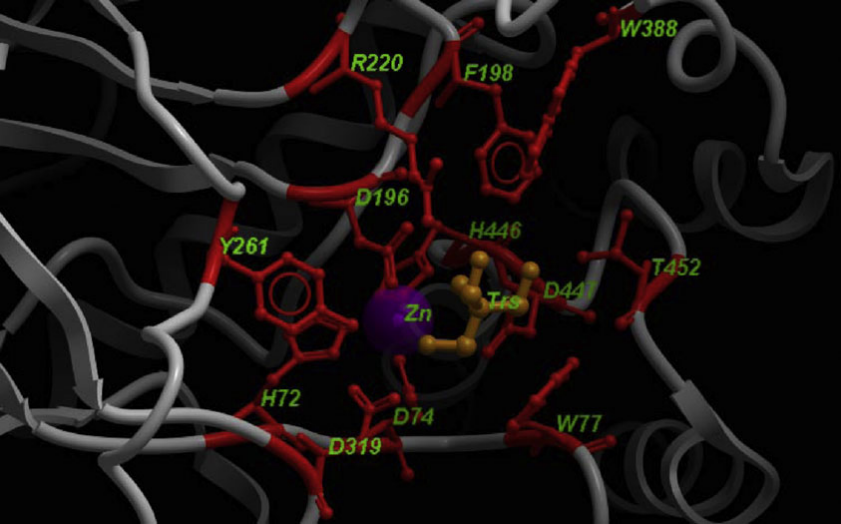
**Active site of bovine lysosomal α-mannosidase**. The Tris ligand (labeled Trs) is shown in orange, the Zn^2+ ^ion in purple and all the active site residues in red (ball-and-stick representation). These active site residues represent a structural hot-spot region, for the lethal phenotype.

Besides the above disease-implicated mutations, other structurally and functionally vital conserved residues could also have fatal consequences when mutated. Specifically, mutations in the residues comprising the active site of the enzyme could have serious effects. Figure 8 shows the Tris ligand, the zinc ion and all the surrounding residues within the vicinity of 5 Å from the ligand, which together form the active site of the enzyme. Therefore, we predict that mutations involving the residues D74, D196, F198, Y261, D319, W388, H446, D447 and T452 (which are the active site residues) can cause extremely compromised enzyme activity and hence lead to severe genotypic and phenotypic expressions. These residues are highly conserved among all the species and are thus capable of severe forms of α-mannosidosis in human, cow, guinea pig and cat upon mutation. This residue set represents a structurally-derived mutation hot-spot, which are otherwise separated along the sequence of α-mannosidase.

### Sequence-based mutational hot-spot regions in the *MAN2B1 *gene

**Figure 9:**
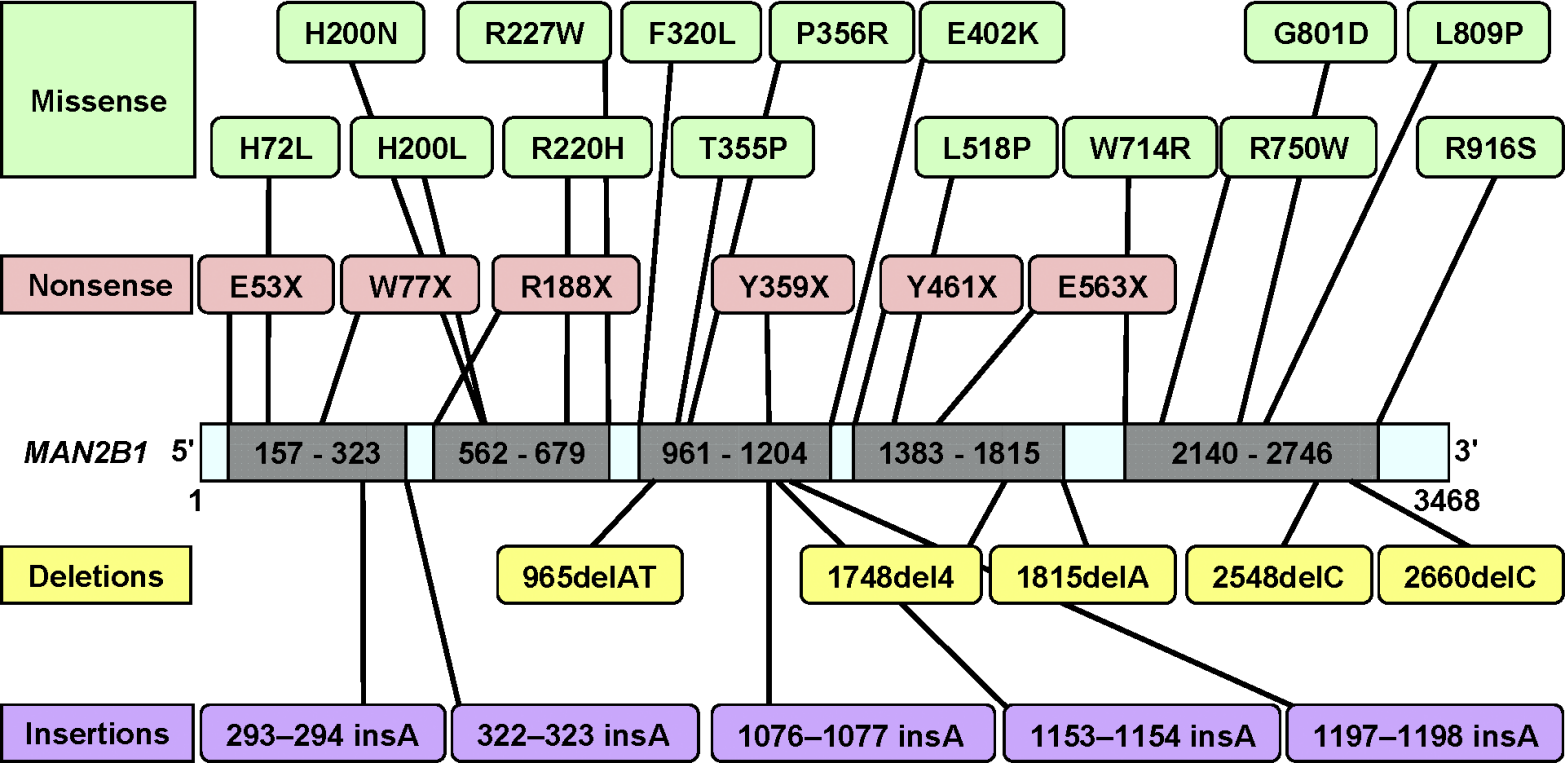
**Location of all non-splicing mutations on the *MAN2B1 *gene sequence**. Missense and nonsense mutations are shown above the gene sequence, with the specific amino acid change indicated, while insertions and deletions are shown below the gene sequence, numbered in terms of the bases affected. The grey areas represent sequence-derived mutational hot-spot regions.

Mapping all the mutations onto the *MAN2B1 *gene sequence revealed the scattering of mutations along the length of the gene. It was also clear that the mutations seemed to cluster in groups over segments of varying sequence length called mutational hot-spot regions, all through the gene. Upon closer investigation it was evident that there were five distinct mutational hot-spot regions with lengths varying from 117 to 606 nucleotides. Figure 9 is a pictorial representation of the mutational mapping to the gene sequence, the nucleotide range of each hot-spot and the concentration of the mutations in every hot-spot. It is inferred that residues coded for by the nucleotides within the range 961-1204 are most likely to undergo mutations due to the large number of mutations found occurring within this range. Residues from other hot-spots are also very likely to be mutated. The hot-spots identified are from nucleotide number 157-323, 562-679, 961-1204, 1383-1815 and 2140-2746. Due to the large number of nucleotides in 1383-1815 and 2140-2746 hot-spot regions the probability of occurrence of a mutational residue coded for by these regions is high. It is also worth noting that the occurrence of a harmful mutation is most likely to be between 157-323, 562-679 and 961-1204 hot-spot regions due to their close proximity to the active site.

## Conclusion

Phenotypically, α-mannosidosis has a range of expression, with the most common manifestations including mental retardation, hearing impairment, skeletal deformities, and recurrent infections. Diagnosis relies on demonstration of deficient α-mannosidase enzyme activity in leukocytes or other nucleated cells such as fibroblasts. From a clinical perspective, variation is considerable, ranging from a severe infantile form that includes profound mental retardation, hepatosplenomegaly, severe dysostosis multiplex and early death to a mild juvenile form that includes moderate mental retardation, hearing impairment, milder dysostosis and survival into adult life. The high phenotypic variability, even between siblings with identical mutations, has so far prevented adoption of a standardized clinical typing, further complicating research into potential treatments.

This analysis establishes a significant correlation between the genotype and the phenotype of the disease. The feline 1748del4 mutation, which causes a severe genotype and an equally fatal phenotype leading to the destruction of the enzyme structure thereby rendering it non-functional, is a good example of our derived relationship. α-mannosidosis caused by this mutation is fatal due to the absence of mannosidase activity in the liver of the Persian cats. There are also missense mutations like the bovine F320L, where the phenotype (with only 0.3% of the normal levels of enzyme activity) was as severe as the effect of the mutation on the genotype of the enzyme. The effect of this mutation cannot be explained by sequence analysis alone. Our work suggests that the mutations in *MAN2B1 *gene are scattered over the entire gene providing us with five mutational hot-spot regions. This gives us an opportunity to predict the degree of severity for a particular mutation and also to predict the residues that are most likely to undergo mutations based on their genotypic location. Moreover, the high degree of mutational heterogeneity of α-mannosidosis is comparable to that observed in many other lysosomal disorders. Based on the co-location of mutations from different organisms (human, cow, guinea pig and cat) and their proximity to the enzyme active site, we have extrapolated observed mutations from one species to homologous positions in other organisms, as a predictive approach for detecting likely α-mannosidosis, based on orthologous positions in the multiple sequence alignment of the α-mannosidase sequences. Our investigation highlights the effect of disease mutations on protein structure and forms the basis for understanding the molecular determinants for phenotypic variations. This study could play a vital role in developing therapies for inherited diseases. Since lysosomal α-mannosidase is an essential enzyme and all observed mutations affect its functioning, our study suggests that rather than drug/inhibitor design, this disease could be tackled through gene therapy.

## Competing interests

The authors declare that they have no competing interests.

## Authors' contributions

JMK carried out the computational simulation studies and drafted the manuscript. JMK and SR participated in the design of the study and interpretation of data. SR developed the project and finalized the manuscript.

## Note

Other papers from the meeting have been published as part of *BMC Bioinformatics *Volume 10 Supplement 15, 2009: Eighth International Conference on Bioinformatics (InCoB2009): Bioinformatics, available online at http://www.biomedcentral.com/1471-2105/10?issue=S15.

## Supplementary Material

Additional file 1 **Table S1**: Non-splicing sequence mutations in the *MAN2B1 *gene Click here for file
