# Supplementary material for: A multi-species comparative structural bioinformatics analysis of inherited mutations in α-D-Mannosidase reveals strong genotype-phenotype correlation
Source: BMC Genomics. 2009 Dec 3;10(Suppl 3):S33. doi: 10.1186/1471-2164-10-S3-S33 (PMC2788387; doi:10.1186/1471-2164-10-S3-S33)
Supplement: Additional file 1 — Table S1: Non-splicing sequence mutations in the MAN2B1 gene [file 1471-2164-10-S3-S33-S1.pdf]

Additional File 1

A multi-species comparative structural bioinformatics analysis of inherited mutations in α-D-Mannosidase reveals strong genotype-phenotype correlation

Javed M. Khan, Shoba Ranganathan

Table S1 - Non-splicing sequence mutations in the MAN2B1 gene

All mutations are those characterized in humans unless otherwise mentioned in parentheses.

No.	Mutation	Sequence change		Effect on coding sequence
		Position	Base	
	**Missense**			
1.	H72L	215	CAT-CTT	His72Leu substitution
2.	H200L	599	A>T	His200Leu substitution
3.	H200N	597	C>A	His200Asn substitution
4.	R220H (cow)	662	G>A	Arg220His substitution
5.	R227W (guinea pig)	679	C>T	Arg227Trp substitution
6.	F320L (cow)	961	T>C	Phe320Leu substitution
7.	T355P	1063	ACC-CCC	Thr355Pro substitution
8.	P356R	1067	CCC-CGC	Pro356Arg substitution
9.	E402K	1204	GAG-AAG	Glu402Lys substitution
10.	S453Y	1358	A>C	Ser453Tyr substitution
11.	L518P	1553	T>C	Leu518Pro substitution
12.	W714R	2140	TGG-CGG	Trp714Arg substitution
13.	R750W	2248	CGG-TGG	Arg750Trp substitution
14.	G801D	2402	G>A	Gly801Asp substitution
15.	L809P	2426	CTG-CCG	Leu809Pro substitution
16.	R916S	2746	C>A	Arg916Ser substitution

No.	Mutation	Sequence change		Effect on coding sequence
		Position	Base	
	**Nonsense**			
17.	E53X	157	G>T	Termination at codon 53
18.	W77X	231	TGG-TGA	Termination at codon 77
19.	R188X	562	C>T	Termination at codon 188
20.	Y359X	1077	TAC-TAA	Termination at codon 359
21.	Y461X	1383	C>G	Termination at codon 461
22.	E563X	1687	GAG-TAG	Termination at codon 563
23.	Q639X	-	CAG-TAG	Termination at codon 639
24.	R760X	-	CGA-TGA	Termination at codon 760
	**Insertions**			
25.	293-294insA	Frame-shift at codon 99		Termination at codon 160
26.	322-323insA	Frame-shift at codon 108		Termination at codon 160
27.	1076–1077insA	Frame-shift at codon 359		Termination at codon 359
28.	1197-1198insA	Frame-shift at codon 399		Termination at codon 438
29.	1153–1154insCC	Frame-shift at codon 384		Termination at codon 476
	**Deletions**			
30.	965delAT	Frame-shift at codon 321		Termination at codon 322
31.	1815delA	Frame-shift at codon 605		Termination at codon 623
32.	1748del4 (cat)	Frame-shift at codon 583		Termination at codon 645
33.	2548delC	Frame-shift at codon 850		Termination at codon 924
34.	2660delC	Frame-shift at codon 887		Termination at codon 932
